# Roles of host proteases in the entry of SARS-CoV-2

**DOI:** 10.1186/s44149-023-00075-x

**Published:** 2023-04-25

**Authors:** Alexandria Zabiegala, Yunjeong Kim, Kyeong-Ok Chang

**Affiliations:** Department of Diagnostic Medicine and Pathobiology, College of Veterinary Medicine, Kansas State University, 1800 Denison Avenue, Manhattan, KS 66506 USA

## Abstract

The spike protein (S) of SARS-CoV-2 is responsible for viral attachment and entry, thus a major factor for host susceptibility, tissue tropism, virulence and pathogenicity. The S is divided with S1 and S2 region, and the S1 contains the receptor-binding domain (RBD), while the S2 contains the hydrophobic fusion domain for the entry into the host cell. Numerous host proteases have been implicated in the activation of SARS-CoV-2 S through various cleavage sites. In this article, we review host proteases including furin, trypsin, transmembrane protease serine 2 (TMPRSS2) and cathepsins in the activation of SARS-CoV-2 S. Many betacoronaviruses including SARS-CoV-2 have polybasic residues at the S1/S2 site which is subjected to the cleavage by furin. The S1/S2 cleavage facilitates more assessable RBD to the receptor ACE2, and the binding triggers further conformational changes and exposure of the S2’ site to proteases such as type II transmembrane serine proteases (TTPRs) including TMPRSS2. In the presence of TMPRSS2 on the target cells, SARS-CoV-2 can utilize a direct entry route by fusion of the viral envelope to the cellular membrane. In the absence of TMPRSS2, SARS-CoV-2 enter target cells *via* endosomes where multiple cathepsins cleave the S for the successful entry. Additional host proteases involved in the cleavage of the S were discussed. This article also includes roles of 3C-like protease inhibitors which have inhibitory activity against cathepsin L in the entry of SARS-CoV-2, and discussed the dual roles of such inhibitors in virus replication.

## Introduction

Coronaviruses are enveloped, single stranded, positive sense RNA viruses and pathogens of humans and a wide variety of animals (Perlman et al. 2020). Since the discovery of avian infectious bronchitis virus (IBV) in 1931 (Seifried 1931), a diverse range of coronaviruses have been reported to cause diseases in humans and animals. In domesticated animals, coronavirus infections cause significant losses to the livestock industry and emotional distress for the owners of critically ill companion animals. Examples of coronaviruses that impact the livestock industry include transmissible gastroenteritis virus (TGEV) and porcine epidemic diarrhea virus (PEDV) in swine (Liu and Gerdts 2021), bovine coronavirus (BCoV) in cattle (Vlasova and Saif 2021), IBV in poultry (Miłek and Blicharz-Domańska 2018) and mink coronavirus in mink (Vlasova et al. 2011). In companion animals, feline (Pedersen et al. 2008), ferret (Provacia et al. 2011), and canine coronaviruses (Licitra et al. 2014; Erles and Brownlie 2008) infect respective species causing local or systemic infections. In humans, four types of respiratory coronaviruses are associated with common cold (HCoV-229E, HCoV-NL63, HCoV-OC43 and HCoV-HKU1). Over two decades ago, in 2002, a novel coronavirus, severe acute respiratory syndrome-associated coronavirus (SARS-CoV), caused outbreaks in Guangdong, China, which then spread to multiple countries (Perlman et al. 2020; Feng et al. 2009).

While SARS-CoV outbreak was contained with its last known case in 2004 (W.H.O. 2004), In 2012, Middle Eastern respiratory syndrome virus (MERS-CoV) emerged in Saudi Arabia (Perlman et al. 2020; Lu and Liu 2012) as a zoonotic disease acquired from dromedary camels. Person-to-person transmission is not efficient with MERS, except for in healthcare settings (Dudas et al. 2018), but mortality rates up to approximately 35% was observed (W.H.O. 2022). MERS-CoV still circulates today, primarily in the Middle East, Africa and southern Asia, where infected dromedary camels transmit the virus to people through direct and indirect contact. Seven years later in 2019, another novel coronavirus, SARS-related coronavirus 2 (SARS-CoV-2) emerged (Huang et al. 2020), causing very mild to severe respiratory symptoms with an elevated mortality in the people with underlying health conditions or older age groups (Ma et al. 2021a; C.D.C.  2022). Unlike its two novel predecessors, SARS-COV-2 spread worldwide, resulting in W.H.O. declaring Covid-19 pandemic in March 2020.

Bat species are considered the major animal reservoir for coronaviruses as well as other viruses that infect mammals (Irving et al. 2021; Latinne et al. 2020), and many coronaviruses infecting humans and animals, including SARS-CoV, MERS-CoV and SARS-CoV-2, can be traced back to them (Irving et al. 2021; Cui et al. 2019; Hu et al. 2017). The expansion of human populations and domestic species has increased the interaction with bat populations (Cui et al. 2019), which is likely to increase the risk of interspecies transmission. Thus, there is a growing concern that novel coronaviruses would periodically emerge and cause outbreaks in human populations (Hu et al. 2017).

Entry of coronavirus into host cells is the key step that determines host susceptibility (Perlman et al. 2020). The spike protein (S) of coronavirus is responsible for viral attachment to the receptor and subsequent entry into its host cells (Perlman et al. 2020). It consists of a trimer and each monomer has two parts, the S1 and S2 region. The S1 contains the receptor-binding domain (RBD) which mediates viral attachment to the host cell functional receptor, while the S2 contains the hydrophobic fusion domain that facilitates viral membrane fusion and entry into the host cells (Perlman et al. 2020). Following the release of the virus from cells, the RBD alternate between open (‘up’ and receptor accessible) and closed (‘down’ and receptor inaccessible), and open RBD binds to the functional receptor such as the angiotensin converting enzyme-2 (ACE2) for SARS-CoV and SARS-CoV-2 (Starr et al. 2020; Lan et al. 2020; Beniac et al. 2007; Yuan et al. 2017). Receptor binding causes a conformational change of the S, resulting in exposure of the S2’ site, and the proteolytic cleavage leads to expose the fusion peptide to cell membrane (Perlman et al. 2020; Gallagher and Buchmeier 2001). The S2’ site and the fusion peptide are well conserved among coronaviruses (Perlman et al. 2020). Mutations in S protein are associated with changes in susceptible animal host, tissue tropism, virulence or pathogenicity (Perlman et al. 2020).

### The role of host protease on the activation of SARS-CoV-2 S protein and virus entry

**Fig. 1:**
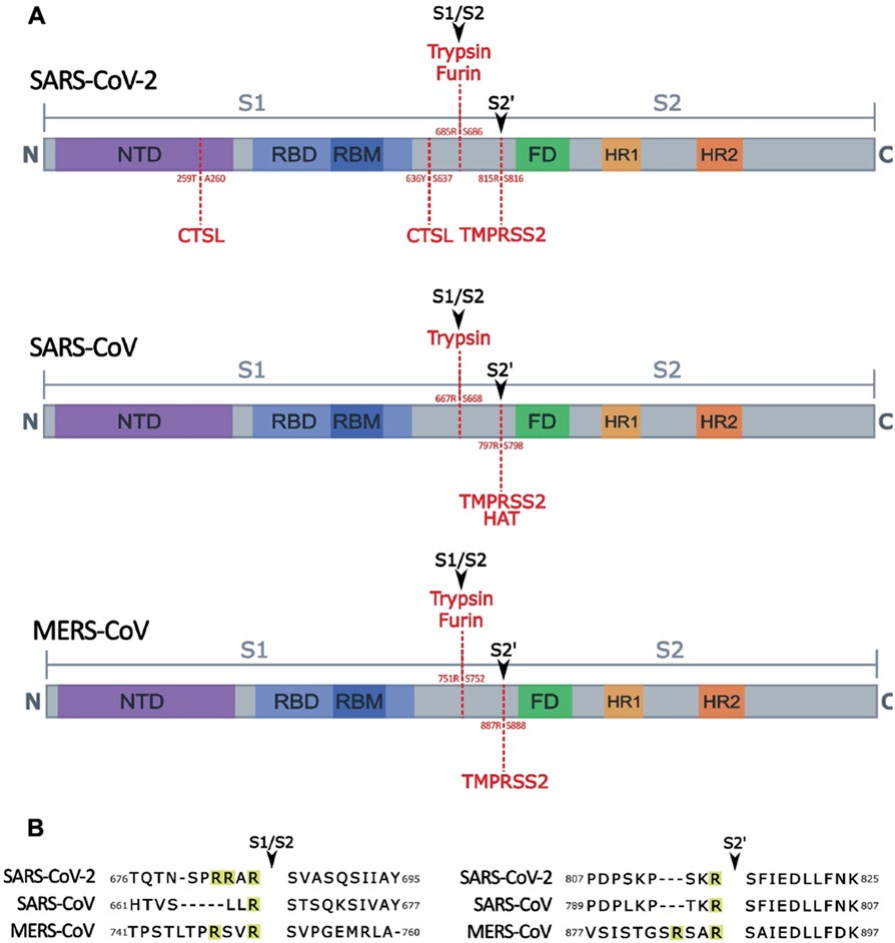
Experimentally observed host protease cleavage sites in S of SARS-CoV-2, SARS-CoV and MERS-CoV. **A.** The S is divided into two portions: S1 and S2. S1 contains the receptor-binding domain (RBD) where the receptor-binding motif (RBM) interacts with the functional receptor (ACE2 or DPP4). The S2 domain contains the fusion domain, which is responsible for viral entry into the cell. In betacoronavirus entry studies, two cleavage sites have been observed as S1/S2 which is cleaved to cause a conformational change to facilitate binding to the functional receptor and S2’ which is cleaved to expose the fusion domain to allow for viral entry. The S2’ site is highly conserved among all coronaviruses and is cleaved by TMPRSS2. (a) In SARS-CoV-2, the S1/S2 cleavage site can be cleaved by host furin and trypsin, and two cathepsin L cleavage sites have been observed in S1 region as pictured. (b) SARS-CoV is unable to be cleaved by host furin but has been observed to be cleaved at the S1/S2 site experimentally by trypsin (Kim et al. 2022b). In addition to TMPRSS2 cleavage at S2’ site, human airway trypsin-like protease (HAT) has also been experimentally observed to have cleavage activity at the site. (c) MERS-CoV S is able to be cleaved by the same proteases as SARS-CoV-2 in the S1/S2 and S2’ sites. **B.** The multibasic arginine (R) resides observed in S of SARS-CoV-2 and MERS-CoV in the S1/S2 region allow for the cleavage of host furin, which shows a specificity for the motif RXXR. S2’ is highly conserved among coronaviruses, as it is responsible for exposing the fusion domain to allow for cell entry

Proteases are one of the most abundant proteins with human and animal genomes encoding approximately 600 proteases (~ 2% of the genomes) (Puente et al. 2003). Proteases are involved in many physiological processes such as digestion, protein activation/turnover, blood coagulation, wound healing, fertilization, cell differentiation and growth, cell signaling, immune response, and apoptosis (Puente et al. 2003). Moreover, proteases are involved in the cleavage and activation of proteins that are produced as proproteins, a process that requires tightly regulation to protect cells from active (enzymatically or structurally) proteins in the wrong places. Therefore, protease activity is controlled by naturally occurring protease inhibitors to prevent uncontrolled, poorly regulated, or undesired protease activity (Turk 2006). Several proteases have been implicated in the activation (cleavage) of SARS-CoV-2 S at various cleavage sites. Summary of host proteases on their distribution, function, cleavage specificity and potential roles in SARS-CoV-2 S activation is listed in Table 1. Figure 1 summarizes overall structures of SARS-CoV-2, SARS-CoV and MERS-CoV S and the cleavage sites of various proteases.

**Table 1:** Summary of host proteases on their distribution, function, cleavage specificity and potential roles in SARS-CoV-2 S activation

Protein	Tissue distribution	Cell distribution	Function	Cleavage P2-P1 / P1’-P2’	S activation	Reference
Furin	Ubiquitous	Golgi body	Cleavage of a wide variety of proproteins into active mature proteins	RXK/RR	S1/S2, RBD (454), S2'(812)	Hoffmann et al. 2020a; Garten 2018; Bergeron et al. 2005; Jaimes et al. 2020; Coutard et al. 2020; Shiryaev et al. 2013; Remacle et al. 2008
Trypsin	Synthesized in pancreas, activated in small Intestine	Intra- luminal	Digestion of proteins in the GI tract	K or R at P1	S1/S2	Woessner et al.^ 2004^; Kim et al. 2022b
Pancreatic elastases	Synthesized in pancreas, activated in small Intestine	Intra-luminal	Digestion of proteins in the GI tract	W, Y, F, L at P1	?	Matsuyama et al. 2005; Belouzard et al. 2010
Neutrophil elastase	Neutrophils	Extra-cellular	Destroys bacteria and localizes neutrophil extracellular traps	W, Y, F, L at P1	thr795	Matsuyama et al. 2005; Voynow and Shinbashi 2021; Kawabata et al. 2002; Kaplan and Radic 2012; Szturmowicz and Demkow 2021)
Matrix metallopeptidase-12 (Macrophage elastase)	Macrophage	Extra-cellular	Breakdown of extracellular matrix	L at P1	?	Shapiro et al. 2004; Guizani et al. 2021
TMPRSS2 tract	Mainly epithelial cells of the lung and prostate. Heart, Liver, and GI tract	Trans-membrane	Digestion, tissue remodeling, blood coagulation, fertility, inflammatory responses	K or R at P1	S2'(812)	Anonymous. 2022; Lam et al. 2015; Koch et al. 2021
HAT	Ciliary respiratory epithelial cells	Trans-membrane	Promotes mucus production	K or R at P1	S1/S2	Takahashi et al. 2001; Chokki et al. 2004; Bertram et al. 2011; Evnin et al. 1990
Matriptase	Oral epithelium, epidermis, Mammary Epithelium	Trans-membrane	Activation of hepatocyte growth factor, hair follicle growth, terminal differentiation of oral epithelium and epidermis	K or R at P1	?	Baron et al. 2013; Oberst et al. 2003; List et al. 2006a; List et al. 2006b; List et al. 2002; Béliveau et al. 2009; Beaulieu et al. 2013
Corin	Cardiac myocytes, uterus	Trans-membrane	Converts pro-ANP to mature ANP (cardiac hormone)	K or R at P1	?	Yan et al. 2000; Bailey et al. 2021; Lindner et al. 2020
Cathepsin B	Widely distributed	Endosome / lysosome	Degrades proteins in endosome/lysosome for recycling. Degrades amyloid beta, activation of pro hormone/enzyme, trypsin activation, activates cathepsin D	G or F at P1	S1 (542), and suggested other cleavage activity	Anonymous. 2022; Yadati et al. 2020; Biniossek et al. 2011; Bollavaram et al. 2021
Cathepsin K		Endosome / lysosome	TLR signaling, processing of B-endorphin in the brain, bone remodeling	Hydrophobic residue in P2	Suggested cleavage activity	Anonymous. 2022; Yadati et al. 2020; Bollavaram et al. 2021; Choe et al. 2006
Cathepsin L	Widely distributed	Endosome / lysosome	Degrades proteins in endosome/lysosome for recycling. Antigen and li chain processing, pheromone processing, controlling neutrophil elastase activity	Hydrophobic aromatic residues at P1	259 & 636, 697?	Anonymous. 2022; Biniossek et al. 2011; Bollavaram et al. 2021; Zhao et al. 2022
Cathepsin S	Widely distributed	Endosome / lysosome	Antigen processing and presentation, li chain processing	Cleavage after basic or hydrophobic residues	Suggested cleavage activity	Anonymous. 2022; Yadati et al. 2020; Biniossek et al. 2011; Bollavaram et al. 2021; Choe et al. 2006
Cathepsin V	Widely distributed	Endosome / lysosome	Natural killer cell and cd8+ cytotoxic cell production	?	Suggested cleavage activity	Anonymous. 2022; Yadati et al.^ 2020^; Bollavaram et al. 2021; Choe et al.^ 2006^

### Protease cleavage sites in S of SARS-CoV-2 and other coronaviruses

The S1/S2 site divides S1 and S2, and Fig. 1B shows the conservation of arginine/serin at the site among SARS-CoV, MERS-CoV and SARS-CoV-2. Most betacoronaviruses except SARS-CoV contain additional arginine residue(s) at the S1/S2 site as shown in Fig. 1B which can be cleaved by furin (Hoffmann et al. 2020a; Garten 2018; Bergeron et al. 2005; Jaimes et al. 2020; Bertram et al. 2013; Coutard et al. 2020). SARS-CoV-2 contains more arginine residues than other known betacoronaviruses with the polybasic cleavage motif (R-R-X-R-R motif) at S1/S2 site (Fig. 1B). The polybasic motif matches the minimal basic motif of RXXR recognized by furin (Garten 2018), a substillin-like protease present in the Golgi apparatus and is involved in the processing of a wide variety of proproteins into their active state (Shiryaev et al. 2013; Remacle et al. 2008). As furin is ubiquitously present in most organs, it has been proposed that the presence of the polybasic motif mediates SARS-CoV-2 entry into different cell types in multiple organ systems. The polybasic site was reported to be associated with virulence and tropism of various viruses, most notably in the hemagglutinin protein of avian influenza (Garten et al. 1994; Stieneke-Gröber et al. 1992; Decha et al. 2008; Horimoto and Kawaoka 1995). The polybasic cleavage site has been directly linked to severe and systemic pathogenesis (Acland et al. 1984; Schrauwen et al. 2012) in bird populations, whereas influenza strains lacking the polybasic cleavage site are typically limited to the respiratory and gastrointestinal tracts (Bertram et al. 2010; Song et al. 2021). The S1/S2 cleavage facilitates more assessable RBD to the receptor ACE2 (Berger and Schaffitzel 2020), and receptor binding causes a conformational change of the S, resulting in exposure of the S2’ site, and the proteolytic cleavage leads to expose the fusion peptide to cell membrane (Perlman et al. 2020; Gallagher and Buchmeier 2001; Millet and Whittaker 2014; Bestle et al. 2020; Papa et al. 2021). The cleavage of the S2’ site allows for shedding of the S1 subunit and HR1 in S2 further undergoes a dramatic refolding transition, which triggers insertion of the fusion peptide into the target cell membrane (Walls et al. 2017; Li et al. 2006; Jackson et al. 2022). In the presence of type II transmembrane serine proteases (TTSPs) such as transmembrane protease serine 2 (TMPRSS2) (Bestle et al. 2020; Glowacka et al. 2011; Shirato et al. 2013; Hoffmann et al. 2020b; Reinke et al. 2022; Belouzard et al. 2009; Vankadari et al. 2022), SARS-CoV-2 can utilize a direct entry route by fusion of the viral envelope to the cellular membrane (Simmons et al. 2005; Bosch et al. 2008). In the absence of this protease, the virus has been shown to utilize an endosomal entry route through processing of the S by endosomal cathepsins (Simmons et al. 2005; Bosch et al. 2008). In SARS-CoV, though exogenous trypsin may have activity at the S1/S2 site in vitro (and gastrointestinal track in vivo), there is limited to no activation by host furin in entry studies of SARS-CoV (Bergeron et al. 2005; Belouzard et al. 2009; Yao et al. 2004; Watanabe et al. 2008).

### TMPRSS2

TMPRSS2 is a transmembrane serine protease primarily expressed in lung and prostate tissue, but is also expressed in a lower amount in heart, liver and GI tract (Anonymous. 2022). The exact physiological function of TMPRSS2 is unknown at this time, as knockout mice showed no phenotypic changes when the protease was not expressed (Kim et al. 2006), though it has been linked to various processes through its role in cancer pathogenesis (Lam et al. 2015). Many studies have shown that the presence of TMPRSS2 is a major determinant of SARS-CoV-2 entry route into respiratory tracts (Koch et al. 2021). The entry of SARS-CoV-2 in various tissues occurs through different pathways depending on the expression levels of TMPRSS2 and other receptors (Jackson et al. 2022; Koch et al. 2021). While it was shown that TMPRSS2 can cleave the S2’ site (Bestle et al. 2020) (Fig. 1), it is uncertain whether other sites such as S1/S2 are processed by TMPRSS2. Some studies indicate that TMPRSS2 acts only on the S2’ cleavage site (Bestle et al. 2020), while others reported that it may have activity at both sites (Reinke et al. 2022; Belouzard et al. 2009). Though the cleavage potential of TMPRSS2 is still debated, it has been demonstrated in SARS-CoV (Glowacka et al. 2011), MERS-CoV (Millet and Whittaker 2014), and SARS-CoV-2 (Koch et al. 2021) that the presence of TMPRSS2 is the determining factor for the entry route utilized, and that cells expressing this protease allow an increased viral entry and replication.

### Cathepsins

**Fig. 2:**
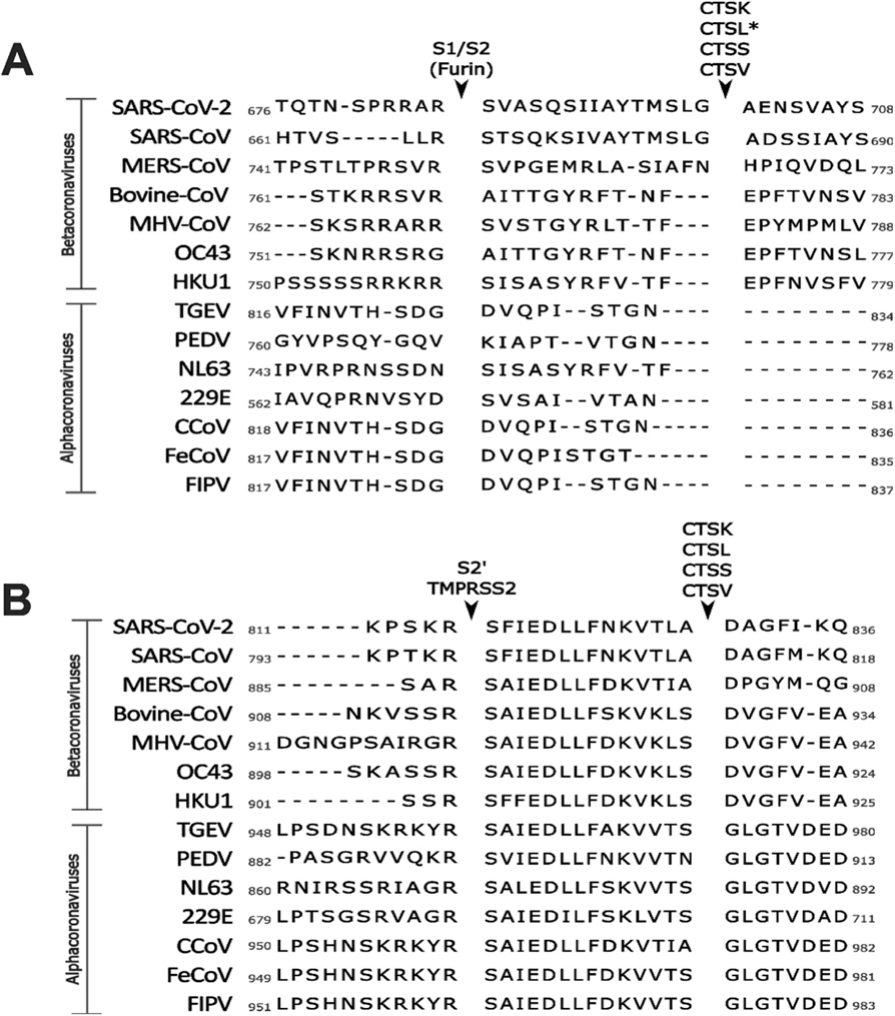
PACMAN prediction of SARS-CoV-2 cathepsin cleavage sites.** A** PACMAN predicted cleavage site of SARS-CoV-2 near S1/S2 and S2’ by cathepsins K, L, S and V aligned with other coronaviruses of importance. *Cathepsin L has also been predicted to cleave at this site in studies of MERS-CoV S. **B** Predicted cleavage site of the cathepsins near the S2’ cleavage site

In the absence of TMPRSS2, SARS-CoV-2, as well as MERS-CoV, can utilize endosomal entry route (Glowacka et al. 2011; Koch et al. 2021; Zhao et al. 2021; Huang et al. 2006; Gierer et al. 2013). A recent report showed that the SARS-CoV-2 Omicron variants may rely more on the endosomal entry than TMPRSS2 mediated cell entry (Willett et al. 2022), suggesting there are variations on the virus entry among SARS-CoV-2 variants. Cathepsins are mainly localized in the endolysosomes and perform a variety of functions such as processing proproteins, hormones and antigens (Yadati et al. 2020; Scarcella et al. 2022). The key cathepsin protease utilized by coronaviruses for cell entry is cathepsin L, a cysteine protease which preferentially cleaves peptide bonds with nonpolar, aromatic residues (Biniossek et al. 2011). It is present in most tissues, including respiratory epithelium, proximal tubules of kidney, cardiomyocytes, and glandular cells of duodenum and colon (Anonymous. 2022). Cathepsin L is also known to activate the entry of various viruses, including Hendra virus and Ebola virus (Chandran et al. 2005; Pager and Dutch 2005). SARS-CoV (Bosch et al. 2008), MERS-CoV (Kleine-Weber et al. 2018), and SARS-CoV-2 have the predicted cleavage sites of multiple cathepsins including cathepsin L and B in the S protein. In SARS-CoV-2 S protein, the cleavage sites are predicted to be at sites near S1/S2 and near S2’ (Bollavaram et al. 2021) (Fig. 2), or cleavage of S protein by cathepsin was observed at two sites in S1 (Fig. 2) (Zhao et al. 2022). While the precise cleavage sites of cathepsin are to be determined, cleavage of S protein upstream of the fusion peptide would allow priming of S2 for fusion of virus and the endosome membrane.

**Fig. 3:**
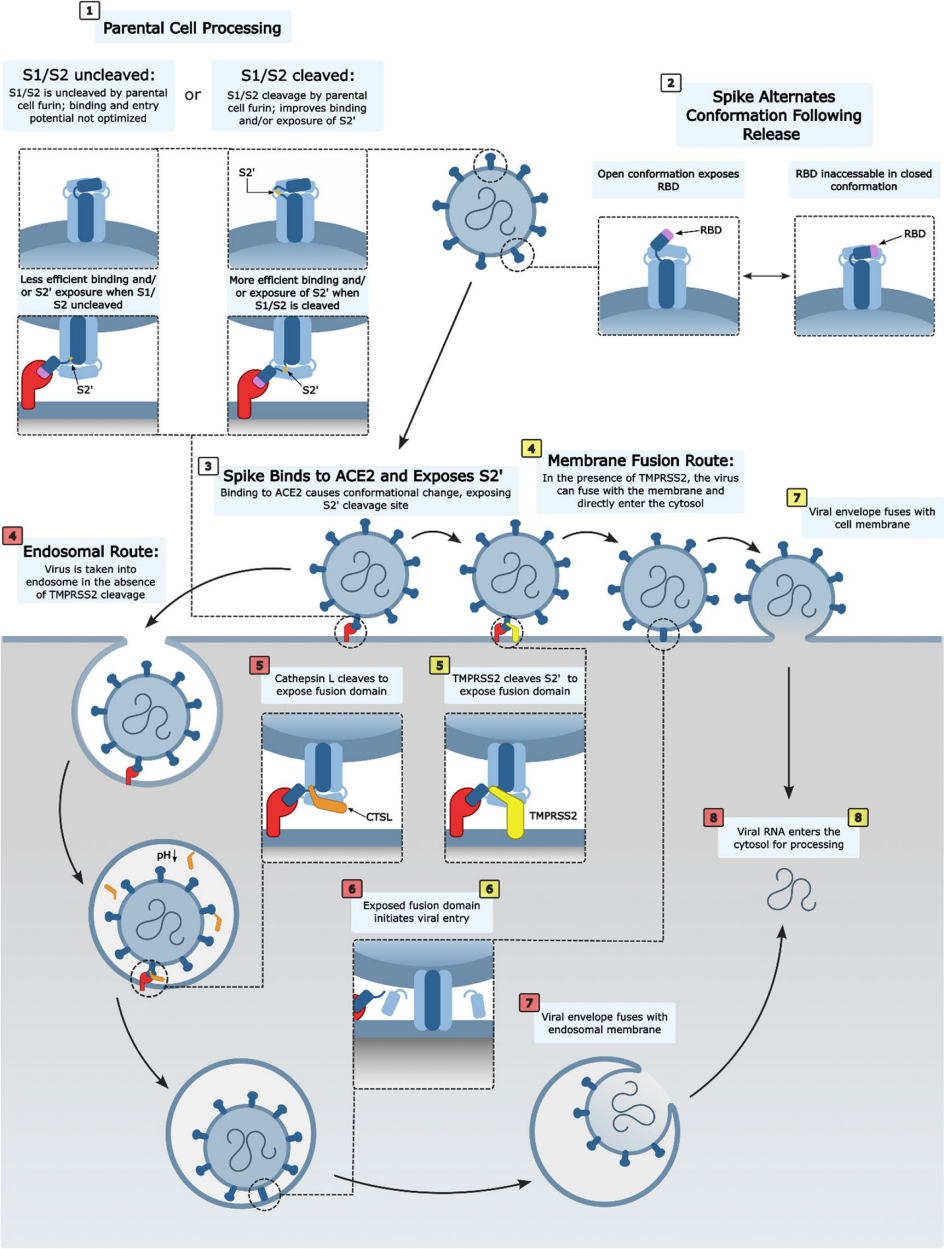
Attachment and entry model of SARS-CoV-2. When SARS-CoV-2 is released by the parental cell, some S is cleaved by host furin. Cleavage by furin facilitates faster binding to the functional receptor ACE2. The binding of ACE2 to S induces a conformational change which exposes the S2’ cleavage site. The presence or absence of TMPRSS2 dictates whether the virus enters through a fast membrane fusion or a slow endosomal route. In the absence of TMPRSS2, the virus is taken into an endosome where the pH will drop, activating cathepsin L. Cathepsin L cleaves S to initiate fusion to the endosomal membrane before release of viral RNA into the cytosol. In the presence of TMPRSS2, the S2’ site is cleaved and the virus can fuse directly to the cell membrane, allowing for a more rapid entry of the viral RNA into the cell

One group utilized a Protease-ase cleavages from MEROPS ANalyzed Specificities (PACMANS) system to predict and rank potential cleavage sites of SARS-CoV-2 S protein, finding high probability of cleavage at sites near S1/S2 and near S2’ (Bollavaram et al. 2021) (Fig. 2). Experimentally, there were two sites observed by one group in S1 for cathepsin L cleavage on SARS-CoV-2 (Fig. 1B) (Zhao et al. 2022). Figure 3 summarizes the entry of SARS-CoV-2 in the presence or absence of TMPRSS2 on the membrane.

Other cathepsins were also reported to be involved in the activation of SARS-CoV-2 S protein. Those cathepsins include cathepsin B (CTSB), cathepsin K (CTSK), cathepsin S (CTSS), and cathepsin V (CTSV) (Bollavaram et al. 2021), all of which cysteine proteases showed a similar preference for cleavage site as cathepsin L (Fig. 2). Cathepsin B is widely distributed throughout the body (The Human Protein Altas. 2022) and is responsible for activation of various proenzymes, prohormones and trypsin (Yadati et al. 2020). It has fairly nonspecific cleavage sites (Biniossek et al. 2011), and had little to no effect on SARS-CoV-2 entry when cathepsin B selective inhibitors were used (Murata et al. 1991; Ou et al. 2020). Cathepsin K is another cysteine protease with a preference of branched hydrophobic residues at the cleavage sites (Choe et al. 2006) and primarily expressed in osteoclasts for bone remodeling but also involved in Toll-like receptor signaling and processing of B-endorphin in the brain (Yadati et al. 2020). Cathepsin S is present in a wide variety of tissues including ciliated cells of the nasopharynx, lung macrophages, and glandular cells in the GI tract and gall bladder (The Human Protein Alas. 2022) and involved in antigen processing and presentation and light chain processing (Yadati et al. 2020). It has fairly nonspecific cleavage activation (Biniossek et al. 2011) but appears to favor branched hydrophobic residues like cathepsin K (Choe et al. 2006). Cathepsin V is widely distributed throughout the body (Anonymous. 2022) and involved in the regulation of natural killer cells and CD8^+^ cytotoxic cells (Yadati et al. 2020). Like cathepsin L, cathepsin V appears to prefer aromatic hydrophobic amino acids (Choe et al. 2006). The current data which suggest the involvement of these cathepsins on the processing of SARS-CoV-2 S protein are mainly based on in silico modelling (Bollavaram et al. 2021). Thus, the roles of these various cathepsins in the activation of coronavirus S protein remains to be determined experimentally.

### Elastase

Elastases are a diverse group of serine proteases that have various functions throughout the body and a wide substrate specificity (Bieth 2001; Hedstrom 2002). Of the elastases, neutrophil elastase is involved in inflammatory lung processes (Matsuyama et al. 2005; Voynow and Shinbashi 2021; Kawabata et al. 2002). Neutrophil elastases are secreted by neutrophils during inflammation and implicated in the formation of neutrophil extracellular traps (NETs), which trap and kill pathogens (Kaplan and Radic 2012). Neutrophil elastases are a prominent protein present in patients' liung with respiratory coronavirus infections and play a role in the pathophysiology of the disease (Szturmowicz and Demkow 2021). *In vitro*, porcine pancreatic elastase treatment of cells increased the entry of SARS-CoV (Matsuyama et al. 2005; Belouzard et al. 2010), which contains an elastase cleavage site in S2 (Belouzard et al. 2010). Our lab reported the adaptation of PEDV in the presence of pancreatic elastase in cell culture (Kim et al. 2017). Macrophage elastase is involved in the breakdown of the extracellular matrix during normal physiologic processes (Shapiro et al. 2004). Macrophage elastase has been evaluated in its role in the pathophysiology of SARS-CoV-2 in lung tissue inflammation (Guizani et al. 2021), but the potential involvement of this elastase in S protein processing have yet to be evaluated. Currently, research of the role of elastases in SARS-CoV-2 infection has primarily focused on the inflammatory process following virus infection, rather than the S protein processing and viral entry.

### Other proteases

There are other proteases that are also implicated in S protein processing of SARS-CoV-2 and other coronaviruses. For example, TTSPs other than TMPRSS2 was reported to be involved in the cleavage of coronavirus S protein. Human airway trypsin-like protease (HAT) is an enzyme that was first isolated from patients with chronic lung disease (Yasuoka et al. 1997) and found to be most prominently in ciliary respiratory epithelial cells (Takahashi et al. 2001). This protease promotes the production of mucus in the respiratory epithelium (Chokki et al. 2004). HAT was shown to cleave the S1/S2 cleavage site of SARS-CoV (Bertram et al. 2011) (Fig. 1) and involved in the cleavage of MERS-CoV S protein (Millet and Whittaker 2014; Park et al. 2016) and influenza virus HA protein (Baron et al. 2013; Böttcher et al. 2006). As a trypsin-like protease, HAT prefers basic amino acids such as arginine and lysine at the cleavage site (Evnin et al. 1990). Thus, this protease may also be able to process SARS-CoV-2 S protein, though this has yet to be investigated. Other TTSPs that have been investigated for SARS-CoV and SARS-CoV-2 include matriptase. Matriptase, which is expressed in most human epithelia (Oberst et al. 2003) and plays an essential role in oral epithelium, epidermis, hair follicles, and thymic epithelium as a barrier (List et al. 2006a; List et al. 2006b; List et al. 2002), is shown to activate virus proteins for entry. Matriptase prefers arginine residue at the cleavage site (Béliveau et al. 2009) and was shown to cleave multiple polybasic sites in influenza HA protein (Baron et al. 2013; Beaulieu et al. 2013). However, Matriptase-3, a related protein that does not cleave influenza HA (Chaipan et al. 2009) did not mediate SARS-CoV entry (Glowacka et al. 2011). Though its substrate specificity implies the potential role of matriptase in cells lacking furin or TMPRSS2, currently there is no data on matriptase activity on coronaviruses. Corin, a TTSP present in cardiac myocytes and involved in blood pressure regulation (Yan et al. 2000), may play a role in SARS-CoV-2-associated myocarditis (Bailey et al. 2021; Lindner et al. 2020), but this has yet to be investigated.

### Inhibitors of host proteases as therapeutic agents for SARS-CoV-2

The involvement of various host proteases in coronavirus entry makes developing therapeutic agents targeting host proteases appealing. Several monoclonal antibodies targeting SARS-CoV-2 S protein have been licensed as preventive or therapeutic antiviral agents for COVID-19 (Jahanshahlu and Rezaei 2020; Wang et al. 2020; Pinto et al. 2020), but substantial decrease in their efficacy against newer virus variants has resulted in FDA revocation of authorization for treatment. Currently, Actemra (Tocilizumab), Molnupiravir and Remdesivir are among antiviral drugs that are approved by FDA for the treatment of COVID-19. As a viral protease inhibitor, Ritonavir-boosted nirmatrelvir (Paxlovid) has received Emergency Use Authorizations from the FDA for the treatment of COVID-19.

Inhibitors of TMPRSS2 (Hoffmann et al. 2020b; Hernandez-Mitre et al. 2022) or cathepsin L (Pišlar et al. 2020) have been evaluated as therapeutic interventions. However, without combination with directly acting antivirals, their therapeutic potential needs to be determined. Even a major protease involved in S processing is inhibited, it is possible that the virus is able to utilize other proteases (TTSPs, elastases or cathepsins) for the infection, and some of them may be upregulated as a result of the inflammatory processes which occur with the disease (Belouzard et al. 2010). Because inflammation is a main feature of COVID-19, inhibitors of host cell proteases associated inflammation have also been explored as treatment for COVID-19 in preclinical studies and clinical trials (Behzadifard and Soleimani 2022; Menendez 2022; Kreidieh and Temraz 2021).

### Effects of various protease inhibitors on SARS-CoV-2 replication in the replicon and virus entry assays in cells

The SARS-CoV-2 3C-like protease (3CLpro) has been a validated therapeutic target with success of Paxlovid in COVID-19 patents, and numerous 3CLpro inhibitors have been shown to be effective in the animal models (mouse and hamster models) as a single or combination treatment (Kuroda et al. 2023; Quan et al. 2022; Abdelnabi et al. 2022; Ma et al. 2021b; Owen et al. 2021; Boras et al. 2021; Caceres et al. 2021; Fu et al. 2020; Qiao et al. 2021; Shi et al. 2021; Vandyck et al. 2021). Some inhibitors of SARS-CoV-2 3CLpro including those from our lab (Dampalla et al. 2021a; Dampalla et al. 2022; Dampalla et al. 2021; Dampalla et al. 2021c; Rathnayake et al. 2020) have dual inhibitory effects against cathepsins (Steuten et al. 2021; Hu et al. 2021; Zhou et al. 2021; Ma et al. 2020). GC376 and calpain inhibitors were shown to inhibit both 3CLpro and cathepsin L in the entry assay using the pseudovirus assay and enzyme assay (Hu et al. 2021). To elucidate the potential dual roles of 3CLpro inhibitors against SARS-CoV-2, we examined selected compounds from our lab on the entry of SARS-CoV-2 using lentivirus-based pseudotyped virus expressing coronavirus S proteins (Kim et al. 2022). We have previously reported the antiviral effects of 3CLpro inhibitors 6e and 6j against SARS-CoV-2 in Vero E6 cells and primary human airway epithelial cells (Rathnayake et al. 2020). In addition to those 3CLpro inhibitors, well known cathepsin L inhibitors including MDL28170 and Z-FL-CHO and a trypsin inhibitor, Nafamostat (all from Sigma-Aldrich, St. Louis, MO) were tested against SARS-CoV-2 3CLpro, cathepsin L, and virus entry and replication. The cathepsin L and 3CLpro inhibition assays were done with cathepsin L inhibitor kit from Abcam (Waltham, MA) and our established assay system (Rathnayake et al. 2020), respectively. The entry inhibition assay with pseudotyped virus expressing SARS-CoV-2 S was performed in 293 T cells expressing ACE2 alone or ACE2 plus TMPRSS2, which was previously established in our lab (Kim et al. 2022). In this assay, the cells were incubated with DMSO (0.1%) or serial dilutions of 6e, 6j, MDL28170, Z-FL-CHO, or Nafamostat, immediately after cells were transduced with pseudotyped virus. The SARS-CoV-2 replicon (Caceres et al. 2021; Dampalla et al. 2021a) was used for SARS-CoV-2 replication assay. The plasmid, pSMART-T7-scv2-replicon (pSMART® BAC V2.0 Vector Containing SARS-CoV-2, Wuhan-Hu-1 Non-Infectious Replicon) (He et al. 2021), was obtained from BEI Resources and the experiments were performed in a BSL-2 setting. The synthetic SARS-CoV-2 replicon RNA was prepared from the pSMART-T7-scv2-replicon as described previously (Caceres et al. 2021; Dampalla et al. 2021a) and electroporated into 293 T cells using the Neon Electroporation system (ThermoFisher, Chicago, IL). After the electroporation, cells were incubated with DMSO (0.1%) or each compound at serially diluted concentrations at up to 50 μM for 30 h, and luciferase activities were measured. The dose-dependent inhibition curve for each compound was prepared for both enzyme and cell-based assays, and the 50% effective concentration (IC_50_ for enzyme assay and EC_50_ for cell-based assay) values were determined by GraphPad Prism software using a variable slope (GraphPad, La Jolla, CA).

Both 6e and 6j were highly potent against SARS-CoV-2 replicon with EC_50_, 0.01 or 0.03 μM, respectively (Table 2). However, neither cathepsin inhibitors (MDL28170 or Z-FL-CHO) nor trypsin inhibitor Nafamostat showed any inhibition against 3CLpro in the enzyme assay or SARS-CoV-2 replicon in the cell-based assay at up to 50 μM (Table 2). Both 6e and 6j have anti-cathepsin L activity with IC_50_ values of 0.03 and 0.05 μM, respectively, which correlated well with inhibition of pseudotyped virus entry in 293 T cells expressing ACE2 alone (Table 2). However, neither 6e nor 6j showed any inhibitory activity in pseudotyped virus entry assay  in 293 T cells expressing both ACE2 and TMPRSS2 at up to 50 μM (Table 2). While both cathepsin inhibitors, MDL28170 and Z-FL170, were highly potent against cathepsin L with EC_50_ 0.01 μM and against pseudotyped virus entry in cells expressing ACE2, they did not show any inhibition in cells expressing both ACE2 and TMPRSS2 up to 50 μM (Table 2). Trypsin inhibitor Nafamostat was highly potent in inhibiting pseudotyped virus entry in cells expressing both ACE2 and TMPRSS2 with EC_50_ 0.001 μM, but it had little effect on pseudotyped virus entry in cells expressing ACE2 alone at up to 50 μM (Table 2). The results show these inhibitors have dual actions against SARS-CoV-2 3CLpro and cathepsin L-mediated pseudotyped virus entry in cells expressing ACE2. However, because most susceptible cells in the respiratory system express both ACE2 and TMPRSS2, the results suggest that cathepsin L inhibition  may not play significant antiviral roles in the major target tissues of SARS-CoV-2 in humans.

**Table 2:** Effects of various protease inhibitors on the 3CLpro and cathepsin L in SARS-CoV-2 replication and entry

	EC50 (μM) Virus replication assay			EC50 (μM) Virus entry assay		IC50 (μM) Enzyme assay	
	Vero E6 cells	HAE (Primary lung cells^a^)	293 T cells with the replicon	293 Tcells with ACE2	293 Tcells with ACE + TMPRSS2	3CLpro	Cath L
6e^a^	0.15 ± 0.71	< 0.5	0.01 ± 0.02	0.03 ± 0.05	> 50	0.17 ± 0.06	0.03 ± 0.02
6j^a^	0.8 ± 0.70	< 0.5	0.03 ± 0.08	0.06 ± 0.08	> 50	0.48 ± 0.08	0.05 ± 0.05
MDL28170	N/T	N/T	> 50	0.01 ± 0.02	> 50	> 50	0.01 ± 0.03
Z-FL-CHO	N/T	N/T	> 50	0.02 ± 0.03	> 50	> 50	0.01 ± 0.02
Nafamostat	N/T	N/T	> 50	> 50	0.001 ± 0.02	> 50	> 50

^a^ 6e and 6j were reported in ref (Rathnayake et al. 2020)

## Data Availability

The data that support the findings of this study are available on request from the corresponding author (KOC).
